# Supraglottic myxoedema successfully treated orally

**DOI:** 10.1530/EDM-23-0078

**Published:** 2024-02-19

**Authors:** Yu Arai, Satoru Okada, Taiju Miyagami, Narumi Sue, Chisato Kainaga

**Affiliations:** 1 Department of Family & General Medicine, Tokyo-Kita Medical Center, Japan; 2 Department of General Medicine, Faculty of Medicine, Juntendo University, Tokyo, Japan

**Keywords:** Adult, Male, Asian - Japanese, Japan, Thyroid, Thyroid, Unique/unexpected symptoms or presentations of a disease, February, 2024

## Abstract

**Summary:**

Myxoedema coma is a severe form of hypothyroidism with multiple organ dysfunction, characterised by an altered state of consciousness and hypothermia. Intravenous thyroid hormone replacement therapy is the preferred treatment for myxoedema. The mortality rate associated with this disease is high, and early detection and intervention are essential. Supraglottal myxoedema is a rare form of periglottic oedema and can be fatal. A previously healthy 66-year-old man presented with impaired consciousness, hypothermia, and nonpitting oedema. Blood tests revealed the presence of hypothyroidism and respiratory acidosis. He was intubated for type 2 respiratory failure; however, severe laryngeal oedema made the procedure difficult to perform. Oral thyroid hormone therapy was initiated under the diagnosis of myxoedema coma. Tracheostomy was performed because of prolonged type 2 respiratory failure and laryngeal oedema. Three weeks after admission, the patient was weaned off the ventilator. Approximately a week later, laryngeal oedema improved, and the tracheostomy tube was removed. The patient was discharged and remained stable for 3 months. This case report describes a patient with comatose myxoedema and supraglottic oedema who was successfully treated with oral medication alone. This case shows that supraglottic oedema should be considered even in the absence of wheezing or other signs of upper airway obstruction.

**Learning points:**

- Myxoedema coma is a differential diagnosis of respiratory acidosis.
- In myxoedematous coma, the possibility of difficult intubation due to supraglottic oedema should be considered.
- Tracheostomy should be considered for supraglottic myxoedema, which often results in prolonged ventilator use.
- Supraglottic myxoedema can be treated with oral medications.

## Background

Myxoedema coma is a severe form of hypothyroidism that can cause multiple organ dysfunction. It is characterised by impaired consciousness and hypothermia. Triggers of myxoedema coma include infection, cerebrovascular disease, cardiovascular events, exposure to cold stimuli, trauma, metabolic disorders, and use of drugs (1). This condition is more common in women and 95% of patients have primary hypothyroidism due to Hashimoto’s disease (2), although central and drug-induced forms have also been reported (1), and Intravenous thyroid hormone replacement is recommended for treating myxoedema coma (3). Early intervention is essential because the mortality rate associated with this condition is approximately 20–25% (4). Supraglottic myxoedema may occur in rare cases of myxoedema coma possibly leading to periglottal oedema and upper airway obstruction (5). This can be fatal.

We describe a male patient who developed supraglottic myxoedema and required tracheotomy after prolonged airway management. The patient was successfully treated with oral thyroid hormones.

## Case presentation

A previously healthy 66-year-old man was admitted to our hospital with a loss of consciousness. The patient had been experiencing oedema of the extremities for a year, which had been in remission and worsening. He was aware of weight gain and dyspnoea on exertion. He was 172 cm tall, weighed 85.7 kg, and had a body mass index of 28.9 kg/m^2^. He had no history of smoking or previous heart failure noted. The patient presented with impaired consciousness, with a Glasgow Coma Scale score of 12 (E3V4M5), a temperature of 35.1°C, a blood pressure of 113/62 mm Hg, a pulse rate of 74 beats/min, a respiratory rate of 16 breaths/min, and an oxygen saturation of 98% (5 L/min). Mild non-pitting oedema of the extremities was noted, although no other abnormalities, such as alopecia or thyroid gland enlargement, were observed on physical examination.

## Investigation

Laboratory findings were as follows: creatine kinase level of 4861 U/L, aspartate aminotransferase level of 198 U/L, alanine aminotransferase level of 165 U/L, creatinine level of 1.34 mg/dL, sodium level of 140 mmol/L, blood glucose level of 104 mg/mL, thyroid-stimulating hormone level of 57.55 (normal range (NR): 0.35–4.94) μIU/mL, free T3 level of <1.25 (NR: 1.71–3.71) ng/mL, free T4 level of 0.42 (NR: 0.70–1.48) ng/mL, anti-thyroid peroxidase antibody level of >600 (NR: <16) IU/mL, and anti-thyroglobulin antibody level of >4000 (NR: <28) IU/mL. Venous blood gas analysis showed respiratory acidosis (pH of 7.219, partial pressure of CO_2_ of 98.7 Torr, bicarbonate level of 40.3 mmol/L). Electrocardiographic findings were normal. Chest radiography revealed cardiomegaly, and echocardiography revealed pericardial effusion without cardiac tamponade. Chest computed tomography revealed an enlarged thyroid gland without airway compression or mild bilateral pleural effusion without lung disease. Examination revealed no findings suggestive of infection.

## Treatment

The patient was intubated in the emergency room and admitted to the intensive care unit for CO_2_ retention due to type 2 respiratory failure. Owing to laryngeal oedema, the patient was intubated with great difficulty. He experienced prolonged severe hypotension requiring vasopressors and mild bradycardia after intubation. Myxoedema coma was diagnosed based on the findings of impaired consciousness, hypothermia, hypotension, bradycardia, type 2 respiratory failure, pericardial effusion, rhabdomyolysis, and hypothyroidism with Hashimoto’s disease. Other than Hashimoto's disease, there were no drugs or infections that could cause myxoedema coma. Treatment was initiated with oral liothyronine (T3)/levothyroxine (T4) (T4: 400 μg initially, then 100 μg/day; T3: 20 μg initially, then 100 μg every 8 h) and hydrocortisone 100 mg every 8 h until adrenal insufficiency was ruled out.

## Outcome and follow-up

After treatment, his consciousness improved, and his hypotension and bradycardia resolved. Tracheotomy was performed on day 15 of admission because of laryngeal oedema and prolonged type 2 respiratory failure caused by central hypoventilation and respiratory muscle weakness. The patient was weaned off the ventilator on day 25 of hospitalisation. Laryngeal oedema persisted for approximately a month. However, the patient showed improvement by day 36 of hospitalisation, and the tracheostomy was closed. He was discharged on day 51 of hospitalisation and was doing well during outpatient follow-up.

## Discussion

This report describes a male patient with supraglottic myxoedema who required tracheotomy after prolonged airway management. The patient was successfully treated with oral thyroid hormones. There are two important points to report in this case.

First, the patient had significant oedema in the upper airway, specifically supraglottic myxoedema, which is rare, and only six cases have been reported. Therefore, its prevalence remains unknown (5, 6). In rats, mucopolysaccharide deposition in the submucosa and connective tissue cells of the larynx has been reported in hypothyroidism (7). Deposition of mucopolysaccharides in the supraglottic region is suggested to cause local oedema (5), resulting in supraglottic myxoedema. Myxoedema coma is more common in older women. In a previous report, four out of six cases of supraglottic myxoedema involved males (5, 6), indicating that it may be more common in males. The treatment of supraglottic myxoedema coma has not been established, and it is unknown whether the use of steroids, commonly used to treat airway oedema during intubation, is effective (5). It takes time for airway oedema to improve, and ventilator management is often required for 2–3 weeks (5). In this case, laryngeal oedema took approximately a month to improve; therefore, a ventilator was used. This case showed the same sex and duration of improvement as previously reported cases of supraglottic myxoedema, suggesting that supraglottic myxoedema may have this characteristic course.

In this case, there were no physical findings suggestive of upper airway obstruction, such as dyspnoea or hoarseness, although intubation was difficult because of laryngeal oedema. In patients with suspected myxoedema coma, it is important to consider the possibility of supraglottic myxoedema, to lower the threshold for intubation, and to have a backup plan for surgical airway management in cases of difficult intubation.

Second, the patient was treated with oral thyroid hormone replacement alone and recovered well despite having supraglottic myxoedema. Most experts recommend intravenous hormone replacement for myxoedematous coma (3) because oral medication absorption is uncertain in cases of gastric atony or ileus associated with hypothyroidism (1). There are reports of disease worsening when switching from intravenous to oral hormone replacement (8). However, there are also reports of no differences observed in mortality between patients receiving oral and intravenous hormone replacement (9). Intravenous hormone replacement is not always readily available in some countries; in our country, it only became available in 2020. Most reported cases of supraglottic myxoedema have been treated with intravenous drugs, whereas very few have been treated with oral drugs. Oral drugs may be effective in countries where intravenous drugs are unavailable or where it is difficult to administer intravenous fluids, such as during home visits.

This case study has some limitations. First, it is unclear whether the bradycardia and hypotension following intubation were induced by myxoedema. In this case, the midazolam was used during intubation. Hence, the possibility of hypotension induced by midazolam cannot be excluded. Similarly dexmedetomidine, which might cause bradycardia, was used in the first few days. However, the bradycardia persisted even after dexmedetomidine was discontinued. Therefore, the bradycardia was considered to be due to myxoedema coma.

Second, prolonged airway oedema may have been caused due to the first intubation. Multiple intubation techniques were required during the initial intubation due to airway oedema. No literature exists on the association between the duration of airway injury recovery and intubation procedures. The use of steroids, which are known to be effective in preventing airway oedema after intubation (10), and a clinical course consistent with the duration of myxoedematous supraglottic oedema suggest that the airway oedema, in this case, was not due to trauma but because of the underlying disease.

Third, oral therapy may have caused prolonged airway oedema and respiratory failure because, as mentioned earlier, there are no reports comparing the use of oral and intravenous medications. Previous reports (5) have shown that even if supraglottic myxoedema is treated with intravenous hormone replacement, the duration of ventilation is long, and tracheostomy is often needed. Hence, it is unlikely that delayed recovery was caused by oral therapy.

## Declaration of interest

The authors declare that no conflict of interest could be perceived as prejudicing the impartiality of the study reported.

## Funding

This research did not receive any specific grant from any funding agency in the public, commercial, or not-for-profit sector.

## Patient consent

Written informed consent for publication of their clinical details was obtained from the patient.

## Author contribution statement

YA, SO, and CK cooperated with the attending physician to care for this patient. YA wrote the manuscript. SO and TM supervised manuscript writing. NS was the attending physician of the patient.
